# Editorial: Quantitative bone imaging methods

**DOI:** 10.3389/fendo.2024.1411512

**Published:** 2024-06-06

**Authors:** Phil Salmon, Fatemeh Alavi, Ali Ghasem-Zadeh

**Affiliations:** ^1^ Bruker Belgium (microCT), Preclinical Imaging, Kontich, Belgium; ^2^ Osteoporosis and Women’s Health research, Toronto General Hospital, University Health Network (UHN), Toronto, ON, Canada; ^3^ Departments of Endocrinology and Medicine, Austin Health, The University of Melbourne, Melbourne, VIC, Australia

**Keywords:** quantitative bone imaging, artificial intelligence (AI), fragility fracture, bone, osteoporosis

## Advancing prediction of fragility fractures with AI-powered quantitative bone imaging

The prediction of fragility fractures has evolved from relying solely on clinical factors and plain x-ray imaging of bones to employing multidimensional approaches, including advanced quantitative bone microstructure imaging, genetics, biomarkers, and other factors. These approaches have enhanced our ability to predict the risk of fragility fractures more precisely, yet we have not fully achieved our goal. The future promises enhanced precision with innovative technologies, insights from large-scale data analysis, and fully automated procedures.

Quantitative bone imaging plays a crucial role in assessing the bone’s macro/microstructure for diagnosing osteoporosis, predicting fragility fractures, guiding preventive strategies, and monitoring and evaluating treatment efficacy and interventions. It serves as a valuable tool for preoperatively assessing bone properties, assisting health professionals in preventing imminent fractures and aiding orthopaedic surgeons in pre-planning procedures for joint replacement, fracture fixation, and spine deformity correction surgeries. This editorial summarises some published studies in the field of bone imaging applications for osteoporosis and arthritis, as well as the incorporation of artificial intelligence (AI) into image analysis.

## Bone imaging in bone remodelling, osteoporosis and arthritis

Bone remodelling is crucial for maintaining skeletal health and integrity. Throughout an individual’s life, all bones—including femoral bones—undergo constant regeneration. This process allows them to adapt to mechanical pressures and maintain structural integrity. Advanced bone imaging techniques offer critical insights into the mechanisms of bone remodelling affects, tracking changes in microstructure and mineralised density. Li et al. conducted a study examining CT scans of the tibial and femoral subchondral bone in the knees of Judo athletes, comparing them with controls. They found that the specific mechanical strains, loads, and impacts associated with this ancient martial art sports led to increased subchondral bone volume and altered bone mineral density distribution. The unique combination of competition bouts and specialised training techniques in Judo, which often stress the joints—especially during puberty—results in significant changes to knee bone structure. These bone mineral density changes are attributed to bone modelling and remodelling, influenced by strains, compressions, hydrostatic pressures, and occasionally, the healing from ligament tears.

In addition to monitoring changes in bone structure and mineral density due to remodelling, bone imaging techniques can also assess the effectiveness of bone treatments. Salmon et al. revisited the mouse model of osteoporosis, focusing on histomorphometric analysis of the trabecular bone at the distal femur and proximal tibia—locations in mouse bones where trabecular bone is extensive enough for analysis. Such analysis, initially conducted via microscopy and more recently by Micro-CT, has become a staple for the translational assessment of trabecular bone outcomes from anti-osteoporosis therapies and genetic phenotypes. This study introduced a dynamic time perspective by considering the bone as fluid in flow, rather than a static structure, unlike existing methods. Profiling morphometric parameters with distance ‘downstream’ from the growth plate has added significant new discriminatory power to bone analysis in mouse studies of low bone mass and drug effectiveness in prevention. It also identified new spatiotemporal trends in the bone’s response to both ovariectomy and treatment, distinguishing between short-term effects near the growth plate and long-term effects further downstream. Essentially, this approach incorporates the time dimension into bone histomorphometry.

The application of imaging techniques like Micro-CT is not limited to bone; it also encompasses conditions like arthritis, which involve quantifying hard and soft tissue at the same time. Oliviero et al. contributed two papers on Micro-CT imaging in mouse models, one examining post-traumatic arthritis through the surgical destabilization of the mouse knee’s medial meniscus. They investigated metaphyseal trabecular bone and subchondral bone in the knee. Oliviero et al. evaluated the accuracy of *in vivo* Micro-CT on anesthetized mice, enabling serial studies where each mouse acted as its own control despite lower image quality from reduced x-ray doses and magnification. Comparing these *in vivo* scans with higher-resolution ex vivo images, they confirmed a strong correlation in morphometric parameters, validating the effectiveness of *in vivo* Micro-CT in these and similar models. However, the sensitivity of low-resolution images in detecting small changes remains questionable.

Reproducibility and the impact of inter- and intra-operator variability are crucial in quantitative imaging. Lower coefficient variation and reduced user-related errors validate the outcomes of image analysis. Oliviero et al. addressed these issues by choosing regions of interest with straighter alignment and less surrounding tissue in the tibia bone of mice. They concluded that reproducibility and inter- and intra-operator variability were acceptable for Micro-CT parameters, showing relatively less error in bone density than in bone morphometrics. The authors concluded that the general reproducibility made Micro-CT suitable for both morphometric analysis and finite element mechanical assessments derived from Micro-CT.

## AI-based image analysis

The integration of AI into bone imaging has revolutionised diagnostics and treatment in endocrinology, orthopaedics and other fields. AI algorithms offer automated, precise evaluations of bone properties by analysing images. AI-driven methods for fracture detection, bone density assessment, and osteoporosis risk prediction enhance diagnostic accuracy, refine treatment strategies, and significantly improve patient care for bone-related conditions.

Bodden et al. demonstrated the significant capability of artificial intelligence to extract novel and clinically valuable data from existing CT scans. Leveraging AI-based neural network techniques, the study analysed lumbar vertebrae volumetric bone mineral density from 420 patients who had undergone thorax and abdomen CT scans for diverse purposes. The findings included odds ratios of 2.2 for existing vertebral fractures and 3.5 for the risk of new vertebral fractures. This research highlights the significant potential of AI analysis for opportunistic screening.

Advancing age deteriorates the composition of the bone mineralised matrix and disrupts the spatial configuration of bone’s three-dimensional architecture. These changes produce a non-linear increase in bone fragility, disproportionate to both the bone loss causing the deterioration and the reduction in bone mineral density. Chapurlat et al. studied the distal radius bone microstructure of 2,666 postmenopausal women, aged 42 to 94 years, using High-Resolution Peripheral Quantitative Computed Tomography. All 3D images were analysed with a deep learning algorithm (DenseNet121 as the feature extraction network) to generate a Structural Fragility Score-Artificial Intelligence (SFS-AI). They reported higher sensitivity and specificity for SFS-AI compared to BMD assessments or FRAX predictions. This study highlights the significance of AI-based image analysis in larger cohorts and AI based segmentation of trabecular and cortical volume of interest masks saved considerable manual effort and time that would be required to do the same analysis without AI.

Bone age assessment in children is a fundamental tool, aiding in the early detection and treatment of growth abnormalities. The integration of AI and automated systems promises to enhance the accuracy and consistency of bone age evaluations, offering a brighter future for paediatric growth assessments. A real-time automated bone age assessment system developed by Yang et al. offers clinicians a valuable and rapid tool for evaluating children’s bone age, which is less labour-intensive and invasive than existing methods.

AI-based bone image analysis has become a reliable method for quantifying iodine uptake in the bone marrow of patients susceptible to cancer. Fervers et al. employed AI for volumetric image analysis, using it to segment marrow volumes in dual-energy CT scans enhanced with iodinated contrast agents, thereby accurately assessing iodine uptake in bone marrow. Valuable baseline data were provided of the iodine uptake in vertebral bone marrow as a function of patient BMD, sex and age.

In addition, Ge et al. have used radiomics- a hierarchical image processing and statistical analysis pipeline, to quantify lumbar spine geometry of patients with fracture who had received vertebral augmentation surgery.

In summary, the incorporation of AI into bone image analysis signifies a significant advancement in the diagnostic and therapeutic approaches to bone, joint, and musculoskeletal-related disorders. This integration offers a more sophisticated and comprehensive framework for treating these conditions. However, further research is essential to fully ascertain the reliability and accuracy of outcomes derived from AI-based image analysis.

## Author contributions

PS: Writing – original draft, Writing – review & editing. FA: Writing – original draft, Writing – review & editing. AG-Z: Writing – original draft, Writing – review & editing.

